# Gene Ontology-based function prediction of long non-coding RNAs using bi-random walk

**DOI:** 10.1186/s12920-018-0414-2

**Published:** 2018-11-20

**Authors:** Jingpu Zhang, shuai Zou, Lei Deng

**Affiliations:** 1 School of Computer and Data Science, Henan University of Urban Construction, Pingdingshan, 467000 China; 2 School of Information Science and Engineering, Central South University, Changsha, 410083 China; 3 School of Software, Central South University, Changsha, 410075 China

**Keywords:** lncRNA, Function annotation, Bi-random

## Abstract

**Background:**

With the development of sequencing technology, more and more long non-coding RNAs (lncRNAs) have been identified. Some lncRNAs have been confirmed that they play an important role in the process of development through the dosage compensation effect, epigenetic regulation, cell differentiation regulation and other aspects. However, the majority of the lncRNAs have not been functionally characterized. Explore the function of lncRNAs and the regulatory network has become a hot research topic currently.

**Methods:**

In the work, a network-based model named BiRWLGO is developed. The ultimate goal is to predict the probable functions for lncRNAs at large scale. The new model starts with building a global network composed of three networks: lncRNA similarity network, lncRNA-protein association network and protein-protein interaction (PPI) network. After that, it utilizes bi-random walk algorithm to explore the similarities between lncRNAs and proteins. Finally, we can annotate an lncRNA with the Gene Ontology (GO) terms according to its neighboring proteins.

**Results:**

We compare the performance of BiRWLGO with the state-of-the-art models on a manually annotated lncRNA benchmark with known GO terms. The experimental results assert that BiRWLGO outperforms other methods in terms of both maximum F-measure (*F*_*max*_) and coverage.

**Conclusions:**

BiRWLGO is a relatively efficient method to predict the functions of lncRNA. When protein interaction data is integrated, the predictive performance of BiRWLGO gains a great improvement.

**Electronic supplementary material:**

The online version of this article (10.1186/s12920-018-0414-2) contains supplementary material, which is available to authorized users.

## Background

The results of the entire human genome sequencing show that only 1.5-2.0% of genes code for proteins. The remaining genes correspond to large non-coding protein regions, which include amounts of transcriptional regulatory elements and non-coding RNA genes. Generally, non-coding RNAs are not capable of encoding proteins [1]. According to the number of bases, non-coding RNAs are divided into long non-coding RNA (lncRNA) and small non-coding RNA (sncRNA). LncRNAs are more than 200 nt in length and highly conserved in their secondary and tertiary structures [2]. With the rapid development of high through-put deep sequencing technology, more and more lncRNAs have been discovered in eukarya in recent years. Especially there is large number of lncRNAs are found in humans and mice [3, 4]. lncRNAs take part in many important regulational processes, such as X chromosome silence, genomic imprinting, chromatin modification, transcription activation, transcription interference, nuclear transport etc [5–7]. Many recent studies have reported that lncRNAs are closely related with occurrence, development, diagnosis and treatment of the disease [8, 9].

With the development of lncRNA research, amounts of data related to lncRNAs emerged. In order to make better use of these information, lots of bioinformatics databases have been built up. These databases contain information about lncRNAs, including structure information, expression information, interaction information of lncRNAs and other relevant information. They play an important role in the research of lncRNAs. Moreover, the data curated by these databases may contribute to research of lncRNAs with computational methods. A brief description of some databases is outlined as follows. NONCODE provides ncRNA related information for 17 species. The information not only includes the basic information of lncRNA such as location, strand, exon number, length and sequence, but also the advanced information such as the expression profiles, conservation info, predicted function and disease relation [10]. LncRNAdb curates the experimentally supported functional lncRNAs [11]. Entries in LncRNAdb are manually curated from referenced literature. ChIPBase aims to explore the transcriptional regulatory networks of ncRNAs and protein-coding genes according to the ChIP-Seq data [12]. lncRNome is a comprehensive searchable biologically oriented knowledgebase for lncRNAs in Humans, which provides various information including chromosomal locations, the types, description on the biological functions and disease associations of lncRNAs [13]. LncRNADisease provides experimentally supported lncRNA-disease associations, which contains approximately 480 entries of high-quality associations [14]. Besides these databases mentioned above, there are still a number of resources about lncRNA, such as GeneCards [15], lncRNASNP [16], lncRNAMap [17], and LncRNA2Target [18] etc.

Although many databases which provide a wide variety of information about lncRNAs have been developed, there are few databases which are focused on function annotation of lncRNAs. Therefore, the functional investigation of lncRNAs has attracted the attentions of many biologists and bioinformaticians [19]. However, sophisticated molecular regulatory mechanisms of lncRNAs remain an enigma. At present, there are still a lot of obstacles to determine the functions of lncRNAs. Biological experiments are the mainly methods to identify the functions of lncRNAs. However, it has the limits with higher cost and time-consuming. In recent years, researchers have developed several computational methods to infer lncRNA functions [20]. Guo et al. [21] proposed a network-based approach, lnc-GFP, to annotate lncRNAs. In lnc-GFP, a bi-colored biological network is built firstly according to co-expression data and protein interaction data, then lncRNAs are annotated by running a global propagation algorithm on the bi-colored network. Jiang et al. [22] developed a method named LncRNA2Function which utilizes hyper-geometric test to predict lncRNA functions. Recently, Zhang et al. [23] calculated the neighboring protein-coding genes of each lncRNA according to the KATZ measure and predicted the functions of lncRNAs in terms of their neighboring genes.

This work is motivated by the promising performance of bi-random walk in predicting the disease-gene association [24, 25] and protein function [26]. In this work, a global network-based approach, BiRWLGO, is proposed to predict potential functions of lncRNAs at large scale. In BiRWLGO, a global network is built by integrating the lncRNA similarity network, the protein-protein interaction (PPI) network and lncRNA-protein associations. Then, the probability score of each lncRNA-protein pair is obtained from applying the bi-random walk algorithm on the global network. Finally, the functions of a query lncRNA can be predicted according to its neighboring proteins. To evaluate the performance of the proposed model, an independent test is performed on the manually annotated 55 lncRNAs with 129 GO terms. Furthermore, we compare the new model with three state-of-the-art models: lnc-GFP [21], LncRNA2Function [22] and KATZLGO [23]. The experimental results show that BiRWLGO achieves F-measure value of 0.345 and outperforms the prediction performance of the other three models. Moreover, case studies also demonstrate the superiority of BiRWLGO on the prediction of the potential functions of lncRNAs.

## Methods

### LncRNA co-expression similarity

The expression profiles of lncRNAs are downloaded from NONCONDE 2016 database [10] that includes the expression profiles of 90062 lncRNAs in 24 human tissues or cells. The evaluation of lncRNA co-expression similarity is conducted by calculating Pearson’s correlation coefficient. And according to the results obtained, we successfully establish the lncRNA similarity network.

### Protein-protein interaction

The PPI data are obtained from STRING V10.0 [27], a database covering data about more than 2000 organisms. The interactions in the database are curated according to high-throughput screening, computational prediction, and information retrieval.

### LncRNA-protein associations

The lncRNA-protein associations are built based on lncRNA-protein interaction data and co-expression data. First, the data about 15941 human lncRNAs and 20284 protein-coding genes from GENCODE Release 24 are downloaded [28]. Then based on the following three sets of data, the genome-wide lncRNA and protein-coding gene associations are obtained:

1. Co-expression data from COXPRESdb [29]
  COXPRESdb reveals the relationships between co-expressed genes in animal species, e.g. human, mouse and fly [28]. From this database, we firstly extract three preprocessed co-expression datasets for human species (Hsa.c4-1, Hsa2.c2-0 and Hsa3.c1-0), including pre-calculated pairwise Pearson’s correlation coefficients (PCC). The correlations are calculated according to the following formula:
  $$ C(l,p)=1-\prod_{k=1}^{K}(1-C_{k} (l,p))\ \ if ~C_{k} (l,p)>0 $$
  Here, *C*(*l*,*p*) represents the overall correlation between lncRNA *l* and protein-coding gene *p*, *C*_*k*_(*l*,*p*) represents the correlation score between *l* and *p* in dataset *k*, and *K* is the number of datasets where *l* and *p* are positively correlated. The gene pairs with negative correlation scores are excluded.
2. Co-expression data from ArrayExpress [30] and GEO
  The co-expression data is extracted from the research of Jiang et al. [22]. The raw RNA-Seq data in 19 human normal tissues are downloaded from ArrayExpress (accession no.E-MTAB-513) and GEO (accession no.GSE30554), respectively. Then, the expression levels of all human lncRNAs and protein-coding genes are calculated through Tophat and cufflinks with the default parameters. The co-expression of lncRNA-protein pairs is evaluated by computing the Pearson’s correlation coefficients.
3. LncRNA-protein interaction data from NPinter [31]
  The known interactions between lncRNAs and proteins are obtained from NPinter v3.0, which contains 491416 experimentally-verified interactions between ncRNAs and other biomolecules. After that, the lncRNA-protein interaction pairs are filtered by restricting the target organisms to “Homo sapiens”. The interactions between lncRNA and protein can be denoted by an binary matrix, each element of which represents whether there is an interaction between an lncRNA and a protein.

### The Gene Ontology annotation

So far, the functions of lncRNAs have not been manually annotated. Hence, in our study, lncRNAs are indirectly annotated according to the existing annotations of proteins. The proteins and their annotations are obtained from the Gene Ontology Annotation (GOA) database [32].

### The BiRWLGO method

**Fig. 1:**
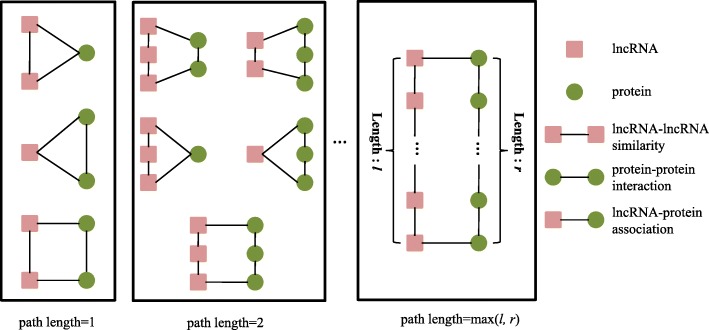
CBGs with different length in the lncRNA-protein association network

A number of methods for predicting the functions of proteins are based on the principle of ’guilty by association’ that a protein tends to exert identical or similar functions with their interacting partners within the protein interaction network. Similarly, the proposed method, BiRWLGO, also exploits the basis. In this work, we annotate lncRNAs according to the known annotations of proteins. Thus, an accurate measurement of the degree of correlation between an lncRNA and annotated proteins is the key for predicting the specific functions of lncRNAs. Furthermore, for measuring the degrees of relevance between lncRNAs and proteins, it is of the first importance to find the mapping between the nodes of the two networks, i.e. lncRNA similarity network and protein interaction network. In the lncRNA similarity network, the adjacent lncRNAs are more possibly mapped to the same protein in protein interaction network. Similarly, proteins that are adjacent in the protein interaction network are likely to be mapped to the same lncRNA in lncRNA similarity network. The correlations among lncRNA-protein associations can be featured by circular bigraph patterns (CBGs) [33, 34]. A CBG is a subgraph that contains an lncRNA path *l*_1_, *l*_2_,⋯, *l*_*k*_ and a protein path *p*_1_, *p*_2_,⋯, *p*_*w*_. The ends of the two paths are connected by two known lncRNA-protein associations. The length of the longer one in the two paths is defined as the length of a CBG (Fig. 1). In reality, CBGs with small lengths can capture most associations in the lncRNA-protein associations. By capturing the CBG patterns with different lengths, the potential lncRNA-protein associations can be revealed.

The bi-random walk approach proposed can be used to discover the lncRNA-protein correlations by capturing the CBG patterns in the lncRNA similarity network and protein interaction network. In the algorithm, the degree of correlation between an lncRNA and a protein is evaluated by its distance to the other associations in the lncRNA similarity network and protein interaction network. Hence, the bi-random walk is a global method to conduct the association map.

**Fig. 2:**
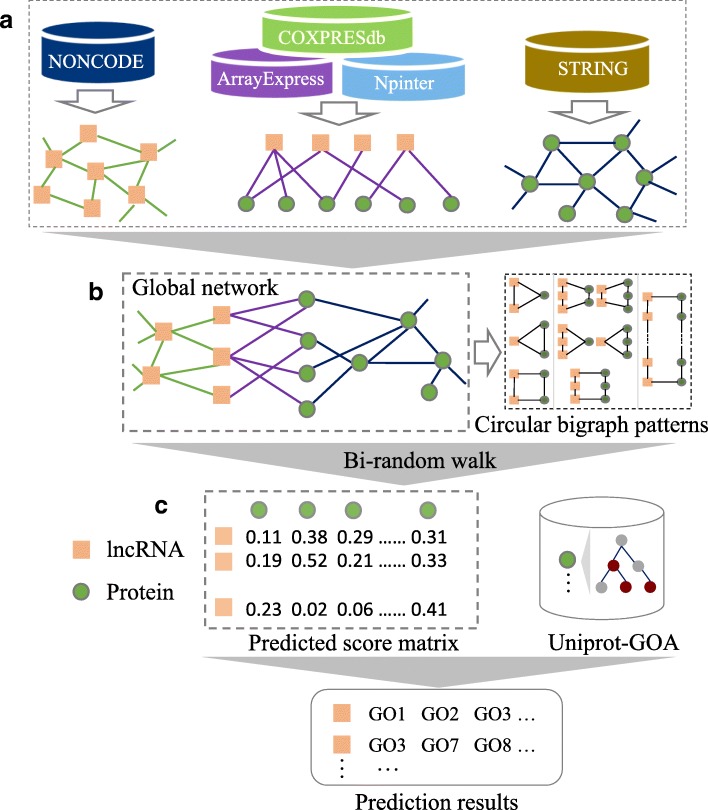
Flowchart of BiRWLGO. It incudes three steps: **a**) build the global network; **b**) run the bi-random walk algorithm on the global network; **c**) annotate lncRNAs with GO terms according to their high ranked neighboring protein-coding genes

Based on the description above, we propose BiRWLGO to annotate lncRNAs by computing the degrees of correlation between lncRNAs and proteins. The flowchart of BiRWLGO is exhibited in Fig. 2. Firstly, a global heterogeneous network consisting of an lncRNA similarity network, a protein interaction network, and lncRNA-protein associations modeled by a bipartite graph is established. Secondly, according to the known lncRNA-protein associations, we run the algorithm of bi-random walk on the lncRNA similarity network and protein interaction network. As a result of the running, the probability scores of association between lncRNAs and proteins are obtained. Finally, the probable functions of lncRNAs are annotated with GO terms according to the high-ranked neighboring protein-coding genes. In the heterogeneous network, *L*(*u*∗*u*), *P*(*v*∗*v*) and *A*(*u*∗*v*) denote the adjacency matrices of the lncRNA similarity network, the protein interaction network and the lncRNA-protein associations respectively, in which *u* represents the number of lncRNAs and *v* represents the number of proteins. Due to the distinct topologies and structures of lncRNA similarity network and protein interaction network, the step numbers of random walk on the two networks might be different from each other. Therefore, the step numbers of random walk on the two sides are restricted by setting two parameters *l* and *r* as the numbers of maximal iterations in the left/right random walk on the two networks. The process of iterative random walk is written as follows:

Left walk on the lncRNA similarity network:

$${R_{L}} = \alpha * {L_{N}} * {R_{t - 1}} + \left({1 - \alpha} \right)A $$

Right walk on the protein interaction network:

$${R_{P}} = \alpha * {R_{t - 1}} * {P_{N}} + \left({1 - \alpha} \right)A $$

Here, *α* refers to the decay factor. *R*_*L*_ and *R*_*P*_ refer to the correlations between lncRNAs and proteins based on the walk on these two networks respectively. Theoretically, the iterative process on two networks could converge to a unique solution and the probability in steady state is defined as the correlation score between an lncRNA and a protein. The algorithm is outlined as Algorithm 1.


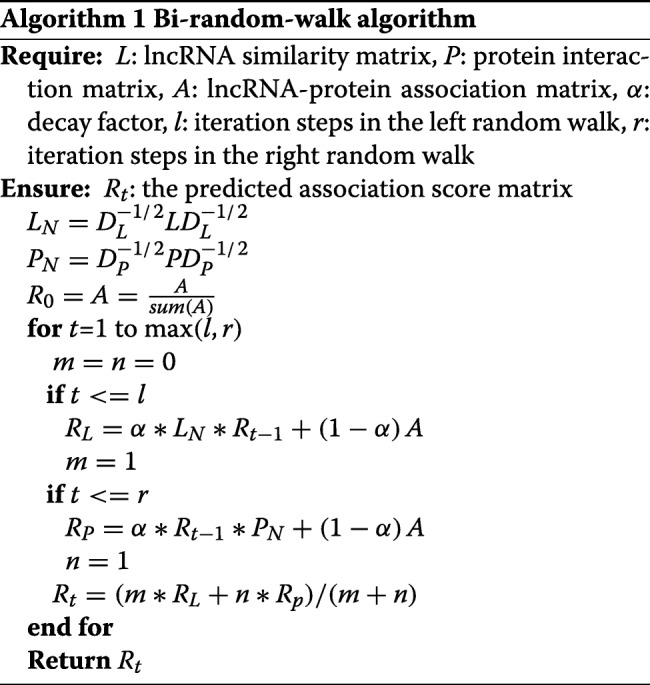


In Algorithm 1, *D*_*L*_ and *D*_*P*_ are both diagonal matrix with diagonal elements ${D_{L}}(i,i){{=}}{\sum }_{j} {{L_{ij}}} $ and ${D_{P}}(i,i){\sum }_{j} {{P_{ij}}} $ respectively. The result of sum(A) is a vector where the entry *i* is defined as ${\sum }_{j} {{A_{ij}}} $. The algorithm will end as it reaches the maximum number of iterations. Finally, the association probability score matrix *R*_*t*_ is acquired, which can represent the relevance probabilities between all lncRNA-protein pairs.

As mentioned above, the functions of a query lncRNA are annotated according to the function information of its top *N* neighboring proteins in a descending order of *R*_*t*_ [35]. The probability *P*_*l*_(*T*_*i*_) for each GO term *T*_*i*_ assigned to the query lncRNA *l* is defined as the sum of weights of neighboring proteins annotated with the term *T*_*i*_:

1 $$ P_{l}(T_{i})=\sum_{i=1}^{N}\frac{S_{lp}(i)}{\sum_{j=1}^{N}S_{lp}(j)} \cdot Ind(T_{i}),  $$

where *S*_*lp*_ represents the correlation score between the query lncRNA *l* and its neighboring proteins *p*, *Ind*(*T*_*i*_) is used to indicate whether a protein is annotated with the term *T*_*i*_. *Ind*(*T*_*i*_) is written as follows:

2 $$ Ind(T_{i}) = \left\{\begin{array}{ll} 1 & if\ I_{i}\ has\ the\ annotation\ T_{i} \\ 0 & otherwise \\ \end{array}\right. {.}  $$

## Results

### Benchmarks

Since the golden-standard dataset of human lncRNA functions has not been established, we first manually annotate 55 lncRNAs with 129 GO terms as the independent test set(lncRNA2GO-55). In lncRNA2GO-55, the lncRNAs are functionally described based on the results from knockdown or overexpression experiments. In these annotations, referenced information about lncRNAs is included, including sequences, structures, genomic context, expression, subcellular localization, conservation, functional evidence etc. The dataset is presented in Additional file 1.

### Evaluation measures

In the proposed model, the output for each term in the GO is a score within [0, 1]. The higher scores indicate more confident predictions. Hence, we introduce a threshold *t* to determine the final predictions. The set of the predicted GO terms is denoted by *P*(*t*), and the set of experimentally determined GO terms is denoted by *T*. The accuracy of prediction is determined by how well the predicted terms match the real ones, which is measured by three well-known statistic metrics, precision (*Pr*), recall (*Rc*) and F-measure (*F*). In this work, for each lncRNA *i* and threshold *t*, the precision and recall are calculated as follows:

$${Pr}_{i}(t) = \frac{\sum_{f \in O} I(f \in P_{i}(t) \wedge f \in T_{i})}{\sum_{f \in O} I(f \in P_{i}(t))} $$

and

$${Rc}_{i}(t) = \frac{\sum_{f \in O} I(f \in P_{i}(t) \wedge f \in T_{i})}{\sum_{f \in O} I(f \in T_{i})} $$

where *f* denotes a GO term and *O* represents the set of GO terms in our experiment. The indicator function *I*(*x*) is written as follows:

$$I(x) = \left\{ \begin{array}{ll} 1 & x=true \\ 0 & x=false \\ \end{array}\right. $$

Given a dataset containing *N* lncRNA-protein pairs, the average precision over a set of *z*(*t*) (≤*N*) lncRNAs on which at least one prediction was made above threshold *t* is defined as:

$$Pr(t) = \frac{1}{z(t)} \cdot \sum_{i=1}^{z(t)} {Pr}_{i}(t). $$

Similarity, the average recall is defined as:

$$Rc(t) = \frac{1}{N} \cdot \sum_{i=1}^{N} {Rc}_{i}(t) $$

on the entire set of *N* lncRNAs.

Large threshold brings about few GO terms being assigned to each lncRNA and results in high precision and low recall. On the other hand, low threshold brings about each lncRNA having many GO terms, and results in high recall and low precision. To solve the problem, we use the maximum F-measure to overall evaluate different methods. The maximum F-measure is written as:

$$F_{max} = {\underset{t}{max}}\left(\frac{2 \cdot Pr(t) \cdot Rc(t)}{Pr(t) + Rc(t)} \right). $$

Moreover, coverage is employed to evaluate these methods. It is defined as the ratio of the portion of lncRNAs which are correctly annotated with GO terms to the whole number of lncRNAs.

### Parameter selection

There are four parameters (*α*, *l*, *r* and *N*) to be tuned in BiRWLGO. The parameter *α* is the decay factor, which is introduced to dampen the importance of a CBG when its path is being longer. The parameters *l* and *r* are employed to limit the number of random walk steps in the lncRNA similarity network and the protein interaction network respectively. A specific lncRNA is annotated according to the GO terms of its top *N* neighboring proteins in *R*_*t*_ in descending order. Therefore, *N* may have an effect on the functional annotations of lncRNAs.

**Fig. 3:**
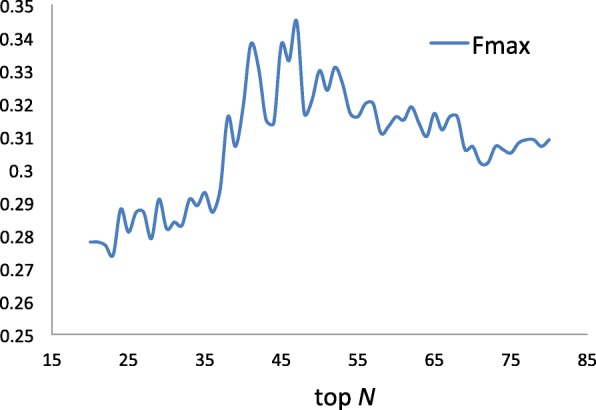
The values of *F*_*max*_ when varying *N* from 20 to 80. The predictive performance of BiRWLGO is sensitive to the actual choice of *N* and the *F*_*max*_ comes to the max value when *N* equals 47

Nevertheless, it is unrealistic to obtain the optimal solution by using exhaustive method. Therefore, in this work, we preset some parameters and then discuss the other parameters. According to other researchers’ work [36], we first set *l* and *r* to (2,2), and then adjust the values of other parameters. First, we calculate the values of *F*_*max*_ when *α* is increasing from 0.2 to 0.9 with step size 0.1. As shown in Table 1, the variation of *α* ranging from 0.2 to 0.7 has little effect on the prediction performance. The *F*_*max*_ values are smaller when *α* ranges from 0.8 to 1. Consequently, we fix *α*=0.8 in the following experiments. Then, we evaluate the performance of BiRWLGO when setting different values of *N* from 20 to 80. The *F*_*max*_ values of BiRWLGO under different assignments to *N* are reported in Fig. 3. The results show that BiRWLGO achieves the best performance when parameter *N* is set to 47. Hence, in our work, we set *N*=47.

**Table 1:** The *F*_*max*_ values when *α* ranges in [0.2, 0.9]

*α*	0.2	0.3	0.4	0.5
*F*_*max*_	0.294	0.305	0.298	0.301
*α*	0.6	0.7	0.8	0.9
*F*_*max*_	0.299	0.300	0.319	0.315

### The effects of protein interaction data

**Fig. 4:**
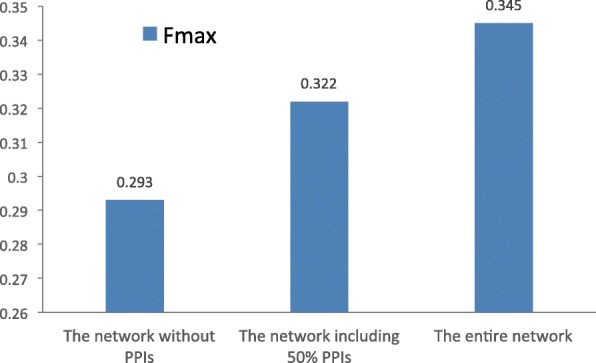
The *F*_*max*_ scores when BiRWLGO is tested on three different network configurations

In our method, we incorporate protein interaction data to help improve the effectiveness of function prediction for lncRNAs. To validate this, BiRWLGO is tested on three different network configurations: the network without PPIs (all PPIs are excluded), the network including 50% PPIs and the entire network (including all PPIs). The performance of BiRWLGO on the three configurations is tested in terms of *F*_*max*_ on the lncRNA2GO-55 dataset. The results are depicted in Fig. 4. The *F*_*max*_ scores are 0.293 for the network without PPIs, 0.322 for the network including 50% PPIs and 0.345 for the entire network. The demonstration reveals that the proposed method can benefit from the integration of the PPI data.

### Performances

Generally, the methods for investigating lncRNA functions are commonly based on ‘guilt-by-association’ from co-expression patterns, namely lncNRAs share similar functions with their protein-coding counterparts [37]. Among these methods, lnc-GFP is aimed to massively annotate the potential functions of lncRNAs. According to gene expression profiles and PPI data, a coding-non-coding bi-colored biological network is established. Then a global propagation algorithm is employed to run on the network to predict the possible functions of unannotated lncRNAs [21]. LncRNA2Function is a statistical approach, which predicts the interested functions according to the correlation between lncRNA expression and expression of protein-coding genes by the hypergeometric test [22]. Recently, Zhang et al. developed a global method, KATZLGO, which can achieve massive prediction of lncRNA functions by integrating multiple biological networks. In KATZLGO, a query lncRNA is annotated according to the GO terms of its neighboring proteins, while the associations between the lncRNA and proteins are calculated based on the KATZ measure [23].

**Fig. 5:**
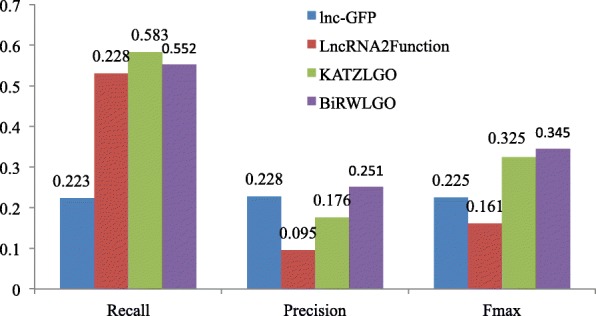
Performance comparison with other methods

To assess the predictive performance of BiRWLGO, we compare it with the three methods described above by an independent test on the lncRNA2GO-55 dataset. GO terms contain three categories, including cellular component, molecular function, and biological process, among which biological process is dominantly emphasized in our experiments for that many well-characterized lncRNAs are involved in the biological process by interacting with proteins and most lncRNAs in lncRNA2GO-55 dataset are annotated with biological terms. The predictive results obtained from different methods on lncRNA2GO-55 dataset are shown in detail in Fig. 5. As shown, our method gains the highest value of *F*_*max*_, which is significantly higher than the other three methods. As for recall, our method also obtains a competitive score of 0.552. Moreover, our method achieves the highest value of precision. Also, we count the number of lncRNAs that are correctly annotated by different methods, and the results are depicted in Table 2. Compared with the other three methods, BiRWLGO correctly annotates 47 lncRNAs, which is the most among the four methods.

**Table 2:** The numbers of lncRNAs correctly annotated by different methods

*Methods*	Unannotated	Annotated
lnc-GFP	22	23
lncRNA2Function	37	18
KATZLGO	10	45
BiRWLGO	8	47

### Case studies

In order to illustrate the prediction ability of BiRWLGO for inferring the potential functions of lncRNAs, we performed case studies in this section. The functions for each selected lncRNAs were confirmed by the literatures.

Case study 1: GHET1. GHET1, gastric carcinoma high expressed transcript 1, is located in an intergenic region on chromatin 7. Yang et al. [38] investigated the biological function of GHET1 in gastric carcinoma. Their results demonstrated that GHET1 promoted gastric carcinoma cell proliferation, specifically increases the stability of c-Myc mRNA and up-regulates its expression. In the clinical analyzing, compared with adjacent tissues, the GHET1 gene and protein expressions were significantly increased in the gastric cancer tissues. In the cell experiment, down-regulation of GHET1 had suppressed the cell proliferation, invasion and migration activities and enhanced the cell apoptosis and G1 phase [39].

To evaluate whether BiRWLGO can functionally annotate the lncRNA GHET1 with functions described above, we apply our method to GHET1. The results show that GHET1 is annotated with 731 GO terms in total. The top 20 GO biological processes are depicted in Table 3. Of the 20 GO biological processes, 8 GO terms are related to regulation as expected. GO:0006417, GO:0042035 and others are involved in the processes that modulate the frequency, rate or extent of the chemical reactions, which have association with cell proliferation. GO:0017148 represents negative regulation of translation, which is consistent with the experimental results in [39]. GO:0010628 is involved in the positive regulation of gene expression, which is demonstrated in [38]. Taken together, the results show that BiRWLGO can successfully predict the functions for lncRNA GHET1.

**Table 3:** The top 20 predicted GO biological process terms for lncRNA GHET1 by BiRWLGO

ID	GO term	GO name
1	GO:0070934	CRD-mediated mRNA stabilization
2	GO:0006417	Regulation of translation
3	GO:0006810	Transport
4	GO:0017148	Negative regulation of translation
5	GO:0010467	Gene expression
6	GO:0051028	Regulation of cytokine biosynthetic process
7	GO:0042035	Regulation of cytokine biosynthetic process
8	GO:0097150	Neuronal stem cell population maintenance
9	GO:0010610	Regulation of mRNA stability involved in response to stress
10	GO:0006403	RNA localization
11	GO:0022013	Pallium cell proliferation in forebrain
12	GO:0006355	Regulation of transcription, DNA-templated
13	GO:0008380	RNA splicing
14	GO:0006397	mRNA processing
15	GO:0000398	mRNA splicing, via spliceosome
16	GO:0007165	Signal transduction
17	GO:0042981	Regulation of apoptotic process
18	GO:0045944	Positive regulation of transcription from RNA polymerase II promoter
19	GO:0010628	Positive regulation of gene expression
20	GO:0001501	Skeletal system development

Case study 2: HOTAIRM1. HOTAIRM1 is located between the HOXA1 and HOXA2 genes and expressed specifically in cells of a myeloid lineage [40]. It can play a regulatory role in myeloid maturation by modulating integrin-controlled cell cycle progression at the gene expression level [41]. In the research of Wan et al. [42], HOTAIRM1 expression was drastically reduced in colorectal cancer tissues compared with matched normal tissues. Moreover, the knockdown of HOTAIRM1 promoted colorectal cell proliferation and over-expression of HOTAIRM1 repressed cell proliferation. It meant that HOTAIRM1 played a role of tumour suppressor in colorectal cancer. Xin et al. [43] demonstrated that HOTAIRM1 competitively bound to miR-3960 and finally regulated the process of hematopoiesis, which revealed a novel regulatory mechanism of lncRNA function.

To examine whether the lncRNA HOTAIRM1 is predicted to have the functions of regulation and differentiation, we apply the method of BiRWLGO to it and find that it is annotated with 271 GO terms. The Table 4 shows the top 20 GO biological processes assigned to the HOTAIRM1. The GO terms in the top 20 include positive regulation and negative regulation, which is in line with the above results. In addition, the term of cell cycle is correctly annotated, which was demonstrated in [41].

**Table 4:** The top 20 predicted GO biological process terms for lncRNA HOTAIRM1 by BiRWLGO

ID	GO term	GO name
1	GO:0006355	Regulation of transcription, DNA-templated
2	GO:0006351	Transcription, DNA-templated
3	GO:0007049	Cell cycle
4	GO:0006397	mRNA processing
5	GO:0008380	RNA splicing
6	GO:0045892	Negative regulation of transcription, DNA-templated
7	GO:0045893	Positive regulation of transcription, DNA-templated
8	GO:0006810	Transport
9	GO:0051260	Protein homooligomerization
10	GO:0016032	Viral process
11	GO:0000398	mRNA splicing, via spliceosome
12	GO:0006366	Transcription from RNA polymerase II promoter
13	GO:0030154	Cell differentiation
14	GO:0045087	Innate immune response
15	GO:0002376	Immune system process
16	GO:0007165	Signal transduction
17	GO:0000122	Negative regulation of transcription from RNA polymerase II promoter
18	GO:0045944	Positive regulation of transcription from RNA polymerase II promoter
19	GO: 0006974	Cellular response to DNA damage stimulus
20	GO: 0001525	Angiogenesis

## Discussion and conclusion

In spite of the fact that a large number of lncRNAs have been discovered over the past decades, only few of them have been functionally described in detail. Since there is lack of conservation and understanding for lncRNAs, it is hard to predict their functions. In this paper, a global network-based strategy, BiRWLGO, is proposed to massively annotate the potential functions of lncRNAs. First, we built a global heterogeneous network based on the data about gene expressions, lncRNA-protein associations, and protein-protein interactions. After that, to obtain the neighboring proteins for each lncRNA, we apply the bi-random walk algorithm on the global heterogeneous network. Finally, a specific lncRNA can be annotated with the GO terms according to its neighboring proteins. In terms of predictive performance, BiRWLGO performs well on the independent dataset lncRNA2GO-55. BiRWLGO acquires the best Fmax score of 0.345. The values of recall and precision are 0.552 and 0.251, respectively. As for coverage, there are 47 correctly-predicted lncRNAs with at least one GO term in the manually-curated 55 lncRNAs. Moreover, the experimental results show that integrating the protein-protein interaction data can improve the performance of function prediction for lncRNAs.

In the future, we can improve BiRWLGO in the following aspects. First, the gene expression data is incomplete, and the reliability is needed to be improved. Embracing more reliable expression data would contribute to the functional annotation for lncRNAs. Secondly, besides the interactions between lncRNAs and proteins, integrating more reliable interactions between lncRNAs and other molecules (e.g. microRNAs) may improve the performance of BiRWLGO. Thirdly, it is well-known that GO functions are organized as a directed acyclic graph hierarchy. Therefore, utilizing the relations among GO terms would increase the power of prediction.

## Additional file

Additional file 1 The lncRNA2GO-55 dataset. Additional file 1 includes the Gene Ontology (GO) annotations and the associated PubMed IDs for 55 lncRNAs. (DOCX 26 kb)
