# Supplementary material for: Gene Ontology-based function prediction of long non-coding RNAs using bi-random walk
Source: BMC Med Genomics. 2018 Nov 20;11(Suppl 5):99. doi: 10.1186/s12920-018-0414-2 (PMC6245587; doi:10.1186/s12920-018-0414-2)
Supplement: Supplementary file 1 — The lncRNA2GO-55 dataset. Additional file 1 includes the Gene Ontology (GO) annotations and the associated PubMed IDs for 55 lncRNAs. (DOCX 26 kb) [file 12920_2018_414_MOESM1_ESM.docx]

**Dataset**

**Table 1.** The lncRNA2GO-55 dataset

| lncRNA | GO term | Pubmed IDs |
| --- | --- | --- |
| tusc7 | GO:0051726 | 23558749 |
| linc00951 | GO:0051726 | 23872665 |
| linc00951 | GO:0070883 | 23872665 |
| ccat2 | GO:0017147 | 23796952 |
| ccat2 | GO:0051726 | 23796952,24504682 |
| PCAT1 | GO:0051726 | 23640607 |
| PCAT1 | GO:0006281 | 21804560,24473064 |
| LUCAT1 | GO:0051726 | 23672216 |
| LSINCT5 | GO:0051726 | 21532345 |
| Xist | GO:0060817 | 8538762,9009199 |
| Xist | GO:0005515 | 18974356,21123648,20542000,21172659,22659877 |
| Xist | GO:0031491 | 20833368 |
| HOTAIR | GO:0045814 | 17604720,20616235 |
| HOTAIR | GO:0032947 | 20616235 |
| HOTAIR | GO:0051726 | 20393566,21637793 |
| LINC00538 | GO:0051726 | 22258142 |
| uca1 | GO:0051726 | 17416635,18501714 |
| IFNG-AS1 | GO:0010468 | 24056746 |
| Jpx | GO:1900095 | 21029862 |
| SNHG3 | GO:0019827 | 21874018 |
| SNHG3 | GO:0003682 | 21874018 |
| SNHG3 | GO:0032403 | 21874018 |
| GACAT1 | GO:0051726 | 23645148 |
| HULC | GO:0035195 | 20423907 |
| HULC | GO:0010468 | 17241883 |
| HULC | GO:0051726 | 17241883 |
| CDR1-AS | GO:0035198 | 21964070 |
| CDR1-AS | GO:0010468 | 23446346 |
| ADAMTS9-AS2 | GO:0010468 | 24833086 |
| ADAMTS9-AS2 | GO:0051726 | 24833086 |
| MALAT1 | GO:0051726 | 19625619,20797886 |
| MALAT1 | GO:0010468 | 20937273 |
| MALAT1 | GO:1990935 | 19116412,20797886 |
| MALAT1 | GO:0043484 | 20797886 |
| MALAT1 | GO:0032403 | 22659877 |
| MALAT1 | GO:0003682 | 22659877 |
| HOTAIRM1 | GO:0010468 | 19144990 |
| HOTAIRM1 | GO:0003682 | 21874018 |
| HOTAIRM1 | GO:0032403 | 21874018 |
| DHRS4-AS1 | GO:0010468 | 22891334 |
| DHRS4-AS1 | GO:0031056 | 22891334 |
| DHRS4-AS1 | GO:0035064 | 22891334 |
| SPRY4-IT1 | GO:0051726 | 21558391 |
| SPRY4-IT1 | GO:0010941 | 21558391 |
| PRNCR1 | GO:0005102 | 23945587 |
| PRNCR1 | GO:0051726 | 23945587 |
| PRNCR1 | GO:0010468 | 23945587 |
| PRNCR1 | GO:0003723 | 23945587 |
| BLACAT1 | GO:0051726 | 23688781 |
| BLACAT1 | GO:0003682 | 23688781 |
| BLACAT1 | GO:0032403 | 23688781 |
| PTCSC3 | GO:0030308 | 22586128 |
| PTCSC3 | GO:0010468 | 22586128 |
| H19 | GO:0001558 | 9203585 |
| H19 | GO:0010468 | 9203585 |
| H19 | GO:0051726 | 7692308,18719115 |
| H19 | GO:0044030 | 9203585,9294195 |
| H19 | GO:0005520 | 10875929 |
| H19 | GO:0003682 | 21172659 |
| H19 | GO:0032403 | 21172659 |
| PVT1 | GO:0051726 | 25043044 |
| PVT1 | GO:0031323 | 2725491 |
| PVT1 | GO:0010468 | 24204837 |
| HOXA11-AS | GO:0050686 | 12050232 |
| WT1-AS | GO:0003723 | 17940140 |
| WT1-AS | GO:0010468 | 17940140 |
| FOXCUT | GO:0010629 | 24889262 |
| FOXCUT | GO:0042127 | 24889262 |
| HOTTIP | GO:0048097 | 21423168 |
| HOTTIP | GO:0003682 | 21423168 |
| LINC00467 | GO:0010468 | 24586304 |
| LINC00467 | GO:0050794 | 24586304 |
| GAS5 | GO:0051726 | 18354083 |
| GAS5 | GO:0001558 | 18354083 |
| GAS5 | GO:0005102 | 20124551 |
| KCNQ1OT1 | GO:0060968 | 18848501,18951091,15516932,21172659 |
| KCNQ1OT1 | GO:0003682 | 18848501,18951091,15516932,21172659 |
| KCNQ1OT1 | GO:0032403 | 18848501,18951091,15516932,21172659 |
| KCNQ1OT1 | GO:0044030 | 20573698 |
| CCAT1 | GO:0042127 | 23143645,24594601 |
| CCAT1 | GO:0051726 | 24594601 |
| LINC00299 | GO:0007420 | 23217328 |
| TUG1 | GO:0061074 | 15797018 |
| TUG1 | GO:0002039 | 19571010 |
| TUG1 | GO:0007049 | 19571010 |
| TUG1 | GO:0003682 | 19571010 |
| TUG1 | GO:0032403 | 19571010 |
| XACT | GO:0016456 | 23334669 |
| NEAT1 | GO:0003682 | 21874018 |
| NEAT1 | GO:0032403 | 21874018 |
| NEAT1 | GO:0006259 | 21170033 |
| LINC00570 | GO:0010628 | 20887892 |
| HEIH | GO:0042127 | 21769904 |
| HEIH | GO:0051726 | 21769904 |
| HEIH | GO:0003682 | 21769904 |
| HEIH | GO:0032403 | 21769904 |
| CRNDE | GO:0019827 | 21874018 |
| CRNDE | GO:0007049 | 21874018 |
| CRNDE | GO:0019222 | 24184209 |
| CCDC26 | GO:0042127 | 16449964 |
| CCDC26 | GO:0045595 | 16449964 |
| CCDC26 | GO:0010941 | 16449964 |
| LINC00970 | GO:0010468 | 23741487 |
| LINC00970 | GO:0030178 | 23741487 |
| ATXN8OS | GO:0003723 | 14972680 |
| ATXN8OS | GO:0043523 | 14972680 |
| MYCNUT | GO:0010468 | 24906397 |
| MYCNUT | GO:0050794 | 24906397 |
| TINCR | GO:0010468 | 23201690 |
| TINCR | GO:0005515 | 23201690 |
| TINCR | GO:0003729 | 23201690 |
| CYP4A22-AS1 | GO:0010468 | 20887892 |
| NPTN-IT1 | GO:0051726 | 23395002 |
| NPTN-IT1 | GO:0042127 | 23395002 |
| NPTN-IT1 | GO:0005515 | 23395002 |
| NPTN-IT1 | GO:0043488 | 23395002 |
| CTBP1-AS | GO:0005515 | 23644382 |
| CTBP1-AS | GO:0010468 | 23644382 |
| CTBP1-AS | GO:0051726 | 23644382 |
| PCGEM1 | GO:0042981 | 16569192 |
| GHET1 | GO:0042127 | 24397586 |
| GHET1 | GO:0043488 | 24397586 |
| GHET1 | GO:0051098 | 24397586 |
| TRERNA1 | GO:0010468 | 20887892 |
| LINC-ROR | GO:0048863 | 21057500 |
| LINC-ROR | GO:0042981 | 21057500 |
| EMX2OS | GO:0010468 | 20066053 |
| DBET | GO:0006338 | 22541069 |
| DBET | GO:0010468 | 22541069 |
